# Emerging discoveries on the role of TRIM14: from diseases to immune regulation

**DOI:** 10.1038/s41420-024-02276-w

**Published:** 2024-12-24

**Authors:** Xinhao Li, Feilong Zhou, Kaiyi Niu, Yizhu Wang, Yanlong Shi, Yunxin Li, Xin Gao, Weijie Zhao, Tianyi Chen, Yewei Zhang

**Affiliations:** Hepatopancreatobiliary Center, The Second Affiliated Hospital of Nanjing Medical University, Nanjing, Jiangsu Province China

**Keywords:** Cancer, Molecular biology

## Abstract

TRIM14 is an important member of the TRIM family and is widely expressed in a variety of tissues. Like other members of the TRIM family, TRIM14 is also involved in ubiquitination modifications. TRIM14 was initially reported as an interferon-stimulated gene (ISG). In recent years, many studies have focused on the regulatory role of TRIM14 in signaling pathways such as the PI3K/Akt, NF-κB, and cGAS/STING pathways and revealed its mechanism of action in a variety of pathophysiological processes, and the regulation of TRIM14 has attracted the interest of many researchers as a new direction for the treatment of various diseases. However, there are no reviews on the role of TRIM14 in diseases. In this paper, we will describe the structure of TRIM14, review its role in cancer, cardiovascular disease, cervical spondylosis, inflammation and antiviral immunity, and provide an outlook on future research directions.

## Facts

- TRIM14, being a specialized member of the TRIM family, lacks the RING structural domain that typically confers E3 ubiquitin ligase activity to family members.
- TRIM14 plays a double-edged role in cancer through multiple mechanisms.
- The abnormal expression of TRIM14 in various diseases such as cancer, cardiovascular and cerebrovascular injuries, and inflammatory diseases implies that it might become an important marker or even a crucial therapeutic target for disease onset and progression.
- The positive function of TRIM14 in antipathogen immunity suggests that it could serve as an effective strategy for the treatment of pathogenic infections, especially viral infections.

## Open questions

- What role does TRIM14 play in cancer and other diseases through which signaling pathways and what are the regulators of its expression?
- By what mechanism does TRIM14 play a role in promoting the body’s immune function against pathogens?
- Through which of its own structural domains do the diverse biological roles of TRIM14 function?

## Introduction

Ubiquitination is a posttranslational modification mediated by the evolutionarily strictly conserved protein ubiquitin (Ub). Ub is covalently coupled to lysine residues on target proteins via E1 (ubiquitin-activating enzyme), E2 (ubiquitin-conjugating enzyme), and E3 (ubiquitin ligase). In addition to serving as a trigger for protein degradation by the 26S proteasome, ubiquitination also plays an important role in apoptosis and necroptosis, selective autophagy, and activation and inactivation of signaling pathways. Thus, ubiquitination can be involved in many pathophysiological states and diseases, such as cancer, inflammation, and infectious diseases [1–4].

The TRIM (TRIpartite Motif) family is a subfamily of RING-containing E3 ubiquitin ligases, most of which contain the characteristic RBCC structure consisting of a really interesting new gene (RING) structural domain, one or two B-box structural domains and a coiled-coil structural domain, as well as a carboxyl-terminal structural domain with highly variable properties, from the N-terminal to the C-terminal [5–7]. To date, more than 80 TRIM family members have been identified. Based on their highly variable carboxyl-terminal structural domains, TRIM proteins containing RING structural domains can be categorized into subfamilies I to XI. Some members cannot be categorized into the above 11 subfamilies due to their lack of RING domains, but they are still regarded as members of the TRIM family because they retain other structural domains (B-box and coiled-coil) in the same order as other members [8, 9]. TRIM family proteins play important roles in immunomodulation, cancer development, and drug resistance through their unique structures and corresponding functions [9, 10].

TRIM14 is an important member of the TRIM family that can activate or inhibit related signaling pathways through interactions with a variety of molecules and plays an important role in various pathophysiological processes. Here, we will first illustrate the structure of TRIM14. Next, we will show the mechanism of its role in tumors, cardiovascular disease, cervical spondylosis, inflammatory response, antiviral immunity, and related regulators. Finally, we will provide a short summary and discuss future research directions related to TRIM14.

## Structure of TRIM14

**Fig. 1:**
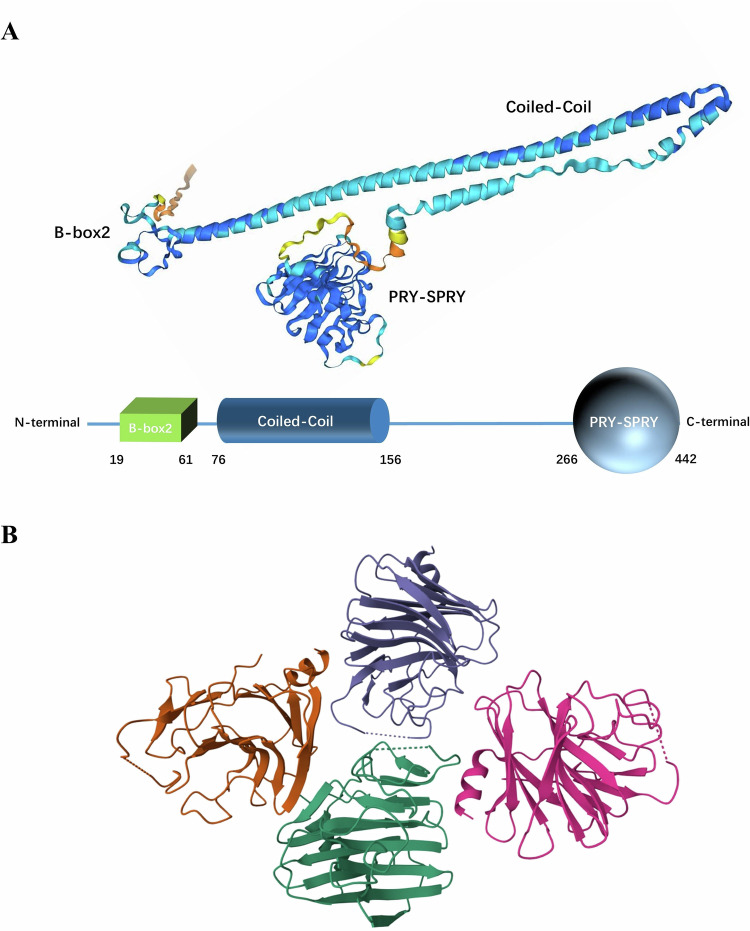
Structure of TRIM14. **A** Molecular 3D structure prediction model made with Alphafold3. **B** The PRY-SPRY domains of the four TRIM14 molecules collectively constitute an asymmetrically organized global unit. Image from the RCSB PDB (RCSB.org) of PDB ID 6JBM (Yu, Y., Liang, L., Jin, Y., Yin, Y., The TRIM14 PRYSPRY domain mediates protein interaction via its basic interface (2019) FEBS Lett 593: 1122–1129).

TRIM14 is a protein composed of 442 amino acids, with a length of 49,773 Da. Its gene is located at 9q22.33. It contains three structural domains: a B-box2 domain, a coiled-coil domain and a PRY-SPRY domain (Fig. 1A). Unlike most other members of the TRIM family, TRIM14 does not contain a RING domain, which makes it a unique member of the family [11].

The B-box domain, which consists of small peptide sequences with finger-like protrusions and contains a “zinc finger” structure, is an important feature of TRIM proteins. Unlike the RING domain, the B-Box domain does not generally exert E3 ubiquitin ligase activity. The B-Box domain has two distinct isoforms, B-Box1 and B-Box2, and in TRIM14, it is B-Box2. The B-Box2 domain is mainly related to the RING and coiled-coil domains and can play a role in ubiquitination. In addition, B-Box2 can participate in protein‒protein interactions and is associated with higher-order self-association, which plays an important role in antiretroviral activity of TRIM5α [12–14].

The CC structural domain is a typical super secondary structure formed by the intertwining of multiple α-helices. In TRIM family proteins, the CC structural domain is involved in homo-multimerization, which is essential for compartmentalization, and this can be illustrated by the fact that the deletion of the CC structural domain causes diffuse localization and prevents the self-binding of TRIMs [15, 16]. In addition, the CC structural domain can also function in association with other structural domains within the molecule. For example, the CC domain and the B-box domain in TRIM5α allow the proteins around the viral capsid to self-associate and thus encapsulate the viral core [17].

The PRY-SPRY (also known as B30.2) structural domain evolved from the SPRY structural domain by the addition of a PRY subdomain, whose core fold is a twisted β sandwich with antiparallel β sheets [18]. Although the PRY-SPRY structural domain has no known enzymatic activity, it can be involved in protein‒protein interactions. It has been reported that the PRY-SPRY structural domain can act as a pattern recognition structural domain, sensitively recognizing the lattice of the retroviral capsid and inhibiting retrotranscription [19, 20]. In addition, the PRY-SPRY structural domain mediates the RNA-binding activity of the protein and is involved in pyroptosis [21, 22].

In TRIM14, the PRY-SPRY domain has been shown to play an important role in antiviral immunity, which can directly interact with viral components to limit viral replication [23, 24]. It has been determined that four PRY-SPRY domains of TRIM14 can form an unsymmetrically arranged whole unit in the form of four molecules (Fig. 1B). This may be due to crystal packing, as the TRIM14 PRY-SPRY protein appears as a monomer in gel filtration assays [11]. However, we can’t rule out that TRIM14 exists as a tetramer. But this requires stronger and more direct evidence.

Although TRIM14 is involved in the ubiquitination of various proteins, the exact mechanism by which this occurs remains controversial. Based on the fact that TRIM44 can interact with TRIM17, which has E3 ubiquitin ligase activity, and inhibit its ubiquitination, an interesting idea has been proposed: TRIM14 binds to and stabilizes other TRIM family proteins with classical RING structural domains through the CC structural domain and co-regulates the ubiquitination degradation of substrate molecules with them [25, 26]. Moreover, although TRIM14 lacks a classical RING structural domain, we should also consider the possibility that TRIM14 has E3 ubiquitin ligase activity, based on the studies of Shen et al. and Sun et al. [27, 28]. It has been shown that other TRIM family protein members that don’t contain RING structural domains can also exert E3 ubiquitin ligase activity through their other structural domains. For instance, in TRIM16 and TRIM29, the B-Box structural domain can replace the RING structural domain and function as the center of the E3 ubiquitin ligase activity for ubiquitination [29, 30]. Therefore, we can hypothesize that TRIM14 may be able to exert its E3 ubiquitin ligase activity through the B-Box structural domain. However, both of these ideas require further experimental proof.

## TRIM14 in disease

Increasing evidence suggests that the irregular expression of many TRIM family proteins play a significant role in the development and progression of diseases. For example, TRIM44 can promote breast cancer progression by enhancing NF-κB signaling and TRIM21 can negatively regulate intestinal mucosal inflammation by inhibiting TH1/TH17 cell differentiation in patients with inflammatory bowel disease [31, 32]. TRIM14, as a member of the TRIM family, also plays an important role in diseases through various signaling pathways and is regulated by multiple factors (Table 1).

**Table 1:** The role of TRIM14 and RNAs associated with its expression in disease.

Type of disease	Function	Mechanism	RNAs associated with regulation of TRIM14 expression	References
Endometrial cancer	Promotion	TRIM14 activates the TBK1/IRF3 signaling pathway and the NF-κB signaling pathway		[38]
Glioma	Promotion	TRIM14 activates the AKT/mTOR/P70S6K signaling pathway and stabilizes Dvl2 to activate the Wnt/β-catenin signaling pathway.	lncRNA CHASERR/miR-6893-3p circ_0005198/miR-198 circ_0000741/miR-379-5p	[44–46]
Breast cancer	Promotion	TRIM14 inhibits SHP-1 and increases p-STAT3 to activate JAK/STAT signaling pathway.	circ_0048764/miR-1296-5p	[47, 132]
Papillary thyroid cancer	Promotion	TRIM14 ubiquitinates SOCS1 to increase p-STAT3 expression.	miR-671-5p miR-4443	[28, 133, 134]
Gastric cancer	Promotion	TRIM14 activates the AKT/mTOR/P70S6K signaling pathway and stabilizes Dvl2 to activate the Wnt/β-catenin signaling pathway.	circ_0091741/miR-330-3p miR-195-5p	[48, 49, 135]
Hepatocellular carcinoma	Promotion	TRIM14 activates the STAT3/HIF-1α signaling pathway.		[52]
Colorectal carcinoma	Promotion	TRIM14 activates the SPHK1/STAT signaling pathway and ubiquitinates PTEN to activate AKT signaling pathway.	lncRNA ELFN1-AS1/miR-191-5p lncRNA GAS6-AS1/miR-370-3p/miR-1296-5p lncRNA MSC-AS1/miR-325	[27, 56, 100, 136, 137]
Oral squamous cell carcinoma	Promotion	TRIM14 activates the NF-κB signaling pathway and is upregulated.	circRNA hsa_circ_0060927/miR-195-5p lncRNA OIP5-AS1/miR-27b-3p lncRNA KCNQ10T1/miR-124-3p miR-195-5p miR-15b	[59–62, 138, 139]
Non-small cell lung cancer	Inhibition	TRIM14 activates IFN signaling and ubiquitinates GFAT1 to inhibit HBP.		[26, 68]
Acute myeloid leukemia	Promotion	TRIM14 activates PI3K/Akt signaling pathway.	miR-23b-5p	[74]
Melanoma	Promotion	TRIM14 activates PTEN/PI3K/Akt and STAT3 signaling pathways.		[72]
Osteosarcoma	Promotion	TRIM14 activates PI3K/Akt signaling pathway.		[73]
Atherosclerosis	Promotion	TRIM14 promotes ox-LDL-mediated vascular endothelial cell dysfunction and facilitates monocyte adhesion to endothelial cells through the NF-κB signaling pathway.	circIRAK1/miR-330-5p circ_0004104/miR-328-3p miR-186-5p	[77–79, 140]
Cerebral ischemia and acute myocardial infarction	Promotion	TRIM14 activates NF-κB/NLRP3 and AKT signaling pathways.		[82, 84]
Cervical spondylosis	Promotion	TRIM14 activates NF-κB signaling pathway.		[86]
Chronic periodontitis	Promotion	TRIM14 activates NF-κB signaling pathway.		[87, 94]
Osteoarthritis	Promotion	TRIM14 activates NF-κB/IFN-β and Wnt/β-catenin signaling pathways.	miR-150-3p	[91, 92]

### The role of TRIM14 in cancer

#### Endometrial cancer (EC)

**Fig. 2:**
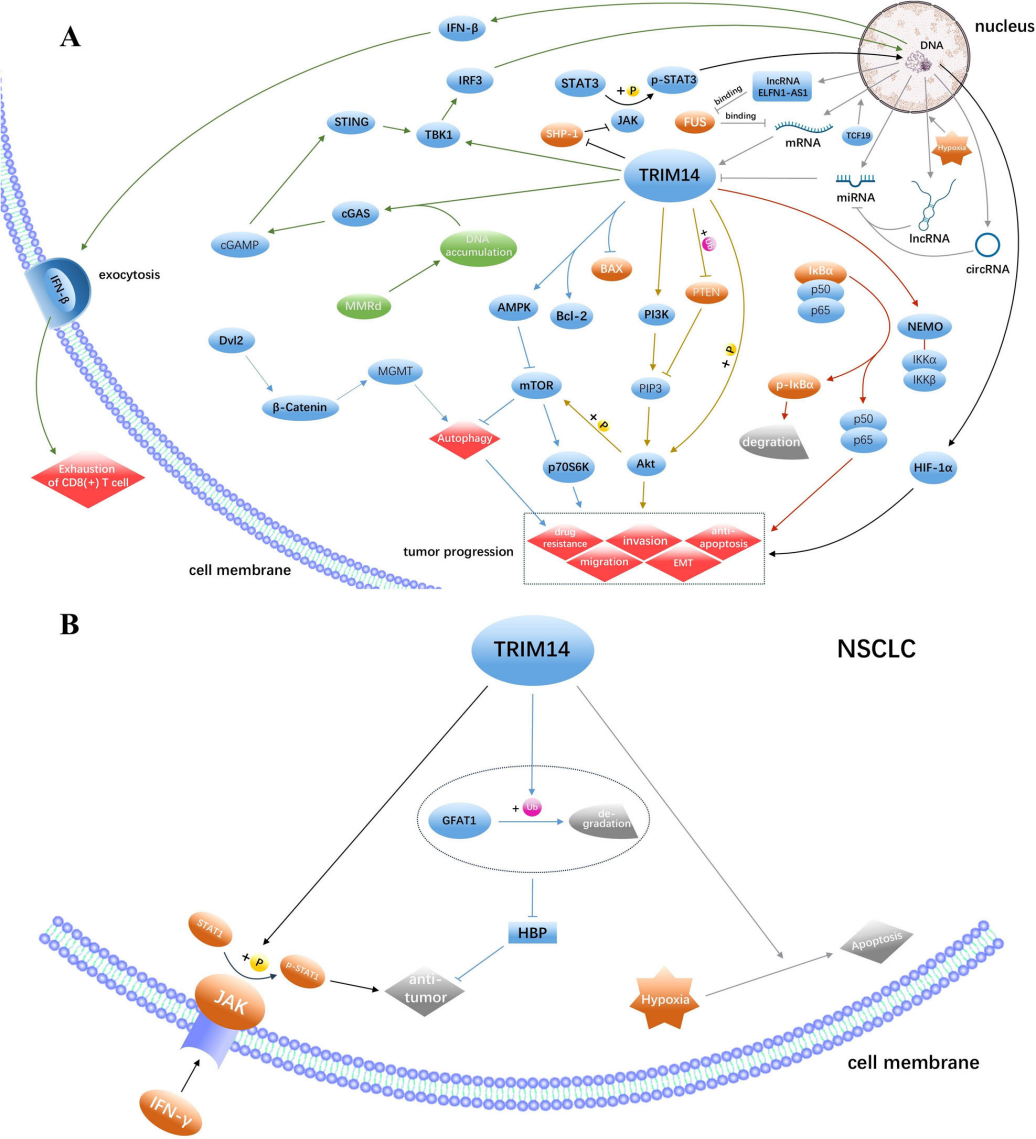
TRIM14 plays a double-edged role in cancer. **A** TRIM14 exerts a pro-cancer effect through various signaling pathways, with its expression being modulated by multiple RNA species. **B** TRIM14 can promote the IFN-γ/JAK/ STAT1 axis, inhibit the GFAT1/HBP axis, and enhance hypoxia-induced apoptosis, thereby functioning as a tumor suppressor in NSCLC.

EC is the sixth most common cancer in women, with the microsatellite instability (MSI) EC subtype, also known as the mismatch repair deficient (MMRd) subtype, accounting for 30% of primary EC cases and 13–30% of late EC cases [33–37]. In MSI-high EC, highly expressed TCF19 can bind the promoter of TRIM14 and promote the expression of TRIM14. TRIM14 then interacts with NEMO to release NF-κB by phosphorylation of IκBα via classical IKK complex, activating the NF-κB pathway and promoting tumor progression. In addition to directly activating TBK1 and recruiting IRF3, TRIM14 synergistically activates cGAS-STING signaling pathway by mismatch repair defect-induced cytoplasmic DNA accumulation, which, in turn, overactivates the TBK1/IRF3 pathway, causing IFN-β to remain at high levels, and sustained IFN-I exposure promotes CD8(+) T-cell exhaustion. These results suggest that TRIM14 may be responsible for promoting cancer and CD8(+)T cell failure of TCF19 by activating TBK1/IRF3 and NF-κB pathways and maintaining high levels of IFN-β [38, 39] (Fig. 2A). And Targeting TRIM14 may improve the tumor microenvironment of EC and delay the progression.

#### Glioma

Glioma is the most prevalent primary tumor of the brain and spinal cord, with glioblastoma (GBM) being the most common and malignant primary brain tumor in adults [40, 41]. TRIM14 expression is low in normal brain tissue and high in glioma specimens, especially in GBM specimens, and its high expression also implies a poor prognosis. It has been shown that TRIM14 can up-regulate and interact with Dvl2 to activate the Wnt/β-catenin signaling pathway, which in turn up-regulates MGMT capable of effectively reversing alkylation-induced DNA damage to promote TMZ resistance in GBM cells [42–44]. Moreover, high expression of TRIM14 may play an important role in promoting resistance to SAHA in GBM cells [45]. In addition, TRIM14 can promote glioma progression by activating the Akt/mTOR/P70S6K and Wnt/β-catenin signaling pathways [46]. Therefore, targeted modulation of TRIM14 may be an effective strategy for ameliorating drug resistance in glioma.

#### Breast cancer (BC) and papillary thyroid cancer (PTC)

TRIM14 expression is reported to be significantly increased in BC and PTC tissues, which also implies a worse prognosis. In BC, TRIM14 can inhibit SHP-1 to increase STAT3 phosphorylation, thus activating the JAK/STAT pathway, downregulating BAX, and upregulating BCL2 to promote cell proliferation and inhibit cell apoptosis [47]. In addition, similar to breast cancer, in PTC, TRIM14 facilitates the ubiquitination and degradation of SOCS1, a negative regulator of STAT3 activation. This promotes the phosphorylation of STAT3, resulting in a decreased BAX/Bcl-2 ratio and inhibition of apoptosis [28]. Accordingly, inhibiting TRIM14 expression in BC and PTC may promote apoptosis and inhibit tumor progression.

#### Gastric cancer (GC)

TRIM14 is expressed at significantly higher levels in GC tissues compared to normal tissues, and this is associated with a poor prognosis. It promotes autophagy by activating AMPK/mTOR pathway, enhances GC resistance to 5-fluorouracil and oxaliplatin, and further promotes GC cell migration, invasion and epithelial mesenchymal transformation through P70S6K. It also activates the Wnt/β-catenin pathway by stabilizing Dvl2, further promoting autophagy and oxaliplatin resistance [48, 49]. Therefore, targeted regulation of TRIM14 may inhibit the progression of GC and improve drug resistance.

#### Hepatocellular carcinoma (HCC)

HCC accounts for approximately 90% of liver cancer cases and is the most common form of liver cancer [50]. TRIM14 expression is significantly elevated in HCC tissues and is associated with poor prognosis in patients with HCC [51]. TRIM14 has the capacity to phosphorylate STAT3, thereby activating the STAT3/HIF-1α signaling pathway and increasing the expression of HIF-1α. This activation results in the promotion of cell proliferation, metastasis, and autophagy in HCC, while concurrently suppressing apoptosis and enhancing resistance to cisplatin chemotherapy [52]. Thus, TRIM14 may be an effective target to inhibit HCC progression and improve drug resistance.

#### Colorectal carcinoma (CRC)

Colorectal carcinoma is the leading cause of cancer-related morbidity and mortality globally, and its incidence and mortality are increasing worldwide [53]. In CRC, the high expression of TRIM14 can induce the proliferation of CRC cells and inhibit apoptosis, which may be achieved by activating AKT pathway through the ubiquitination of PTEN by TRIM14 [27]. In addition, TRIM14 promotes the expression of SPHK1 in CRC cells, which can catalyze the formation of Sphingosine 1-phosphate, thereby inducing constitutive activation of STAT3 and promoting tumor angiogenesis, growth, and metastasis [54–56]. Thus, TRIM14 may be a marker for CRC metastasis, and inhibition of TRIM14 expression may also help limit CRC metastasis [54–56].

#### Oral squamous cell carcinoma (OSCC)

Conventional oral squamous cell carcinoma is one of the most common cancers of the head and neck, occurring in the oral cavity and oropharynx, with the oral tongue being the most common subsite [57, 58]. It has been reported that high expression of TRIM14 in oral leukuplakia in oral leukoplakia is associated with an increased risk of progression to OSCC [59]. In oral tongue squamous cell carcinoma (TSCC), TRIM14 expression is significantly upregulated and can promote TSCC progression through the NF-κB signaling pathway, leading to a poorer prognosis [60]. Elevated TRIM14 expression is also involved in the formation of cancer-initiating cells and epithelial-mesenchymal transition, and is strongly associated with resistance to DDP in TSCC [61, 62]. Consequently, TRIM14 could serve as a biomarker for the malignant transformation of oral leucoplakia, and its inhibition may improve OSCC treatment outcomes.

#### Non-small cell lung cancer (NSCLC)

Interestingly, unlike its expression in many other tumors, TRIM14 is expressed at significantly lower levels in NSCLC than in paraneoplastic tissues. And overexpression of TRIM14 inhibits the proliferation and migration of NSCLC cells. Moreover, TRIM14 interacts with GFAT1, a key rate-limiting enzyme in the hexosamine biosynthesis pathway (HBP), and promotes its ubiquitination degradation, thereby inhibiting HBP and playing a tumor-suppressive role [26, 63–66]. In addition, TRIM14 can positively regulate signaling of type II interferon (IFN) in lung cancer cells, which is an effective factor for most effector cells involved in antitumor immune responses. In this process, TRIM14 mediates the phosphorylation and activation of STAT1 after IFN-γ stimulation, and the modified STAT1 can widely improve IFN signaling, thus functioning as a tumor suppressor. Besides, TRIM14 can also enhance the response to hypoxia-induced cell death [67–71] (Fig. 2B). It is evident that TRIM14 may play diametrically opposite roles in different cancers. And promoting the expression of TRIM14 is expected to inhibit the progression of NSCLC. But why TRIM14 does not exist as a cancer-promoting factor, as it does in other cancers, is a question worth pondering.

#### Other cancers

It has been reported that TRIM14 expression is significantly elevated in acute myeloid leukemia, melanoma, and osteosarcoma, all of which can promote tumorigenesis and progression by activating the PI3K/Akt pathway. In addition, in melanoma, TRIM14 can also activate the STAT3 pathway to exert its pro-oncogenic effect [72–74]. These research results provide a new idea for the treatment of these cancers.

### TRIM14 in cardio-cerebrovascular lesions and cervical spondylosis

#### Atherosclerosis

Atherosclerosis remains a major threat to human vascular health worldwide, and oxidized low-density lipoprotein (ox-LDL) is closely related to the pathogenesis of Atherosclerosis [75, 76]. In ox-LDL-treated human umbilical vein endothelial cells and cardiac microvascular endothelial cells, upregulated TRIM14 promotes ox-LDL-mediated vascular endothelial dysfunction [77, 78]. In addition, a study revealed that the expression of TRIM14, which has a positive regulatory effect on endothelial cell activation by activating the NF-κB signaling pathway, can be significantly induced by inflammatory cytokines such as TNF-α, IL-1β, and LPS. Under TNF-α stimulation, TRIM14 promotes the phosphorylation of p65 through the NF-κB signaling pathway, and activated p65 can directly bind to the promoter region of the human TRIM14 gene to promote transcription. Overexpression of TRIM14 significantly increased the expression of adhesion molecules and cytokines, such as TNF-α, in activated endothelial cells, thus promoting monocyte adhesion to endothelial cells [79]. These findings suggest that TRIM14 may play an important role in ox-LDL-mediated endothelial cell injury and AS progression, and is associated with coronary artery disease.

#### Cerebral ischemia and acute myocardial infarction

Cerebral ischemia and acute myocardial infarction are both diseases with high clinical mortality rates. Early and successful reperfusion is considered to be a very effective strategy, but subsequent reperfusion injury can still adversely affect clinical outcomes [80, 81]. TRIM14 expression is reportedly elevated in both the myocardium and brain after ischemia/reperfusion injury. Inhibition of TRIM14 attenuates brain injury after ischemia/reperfusion, suppresses neuronal apoptosis and inflammatory responses, and ameliorates cognitive dysfunction, partly through modulation of the NF-κB/NLRP3 pathway [82, 83]. Furthermore, in the heart, the overexpression of TRIM14 promotes cardiomyocyte hypertrophy and fibrosis and positively regulates stress-load-induced cardiac hypertrophy and cardiac insufficiency, possibly through the activation of the AKT signaling pathway by TRIM14 [84]. As described above, TRIM14 plays an important role in cardiovascular lesions.

#### Cervical spondylosis

**Fig. 3:**
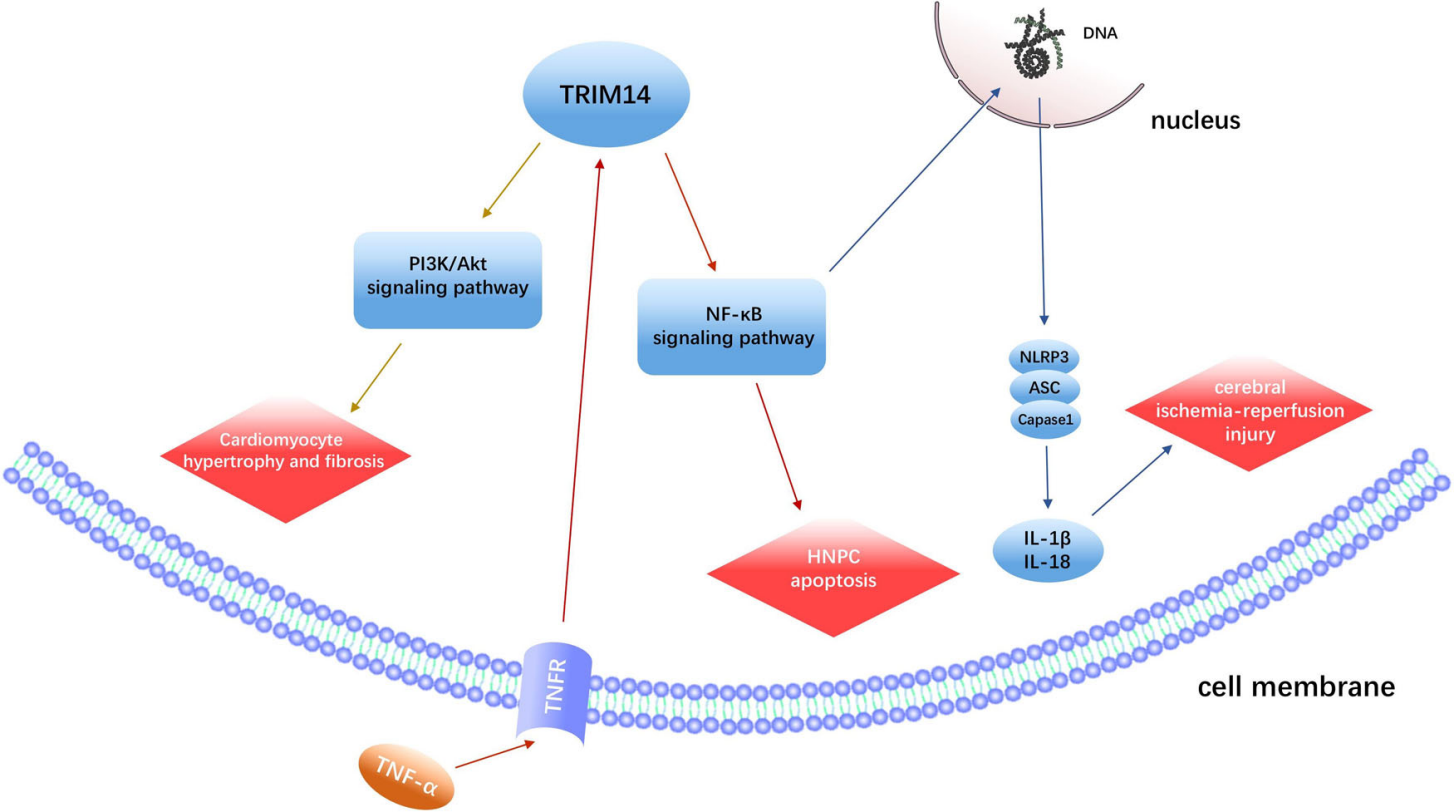
TRIM14 plays an important role in cardiovascular disease and cervical spondylosis through PI3K/AKT, NF-κB signaling pathway and a variety of cytokines.

It has been reported that TNF-α expression is elevated in intervertebral disc degeneration and cervical spondylosis. In TNF-α-induced human nucleus pulposus cells, TRIM14 expression is significantly upregulated and further promotes NF-κBp65 expression and apoptosis in human nucleus pulposus cells, while TRIM14 inhibition has the opposite effect. This provides a possible target for the treatment of cervical spondylosis [85, 86] (Fig. 3).

### TRIM14 promotes inflammatory response

**Fig. 4:**
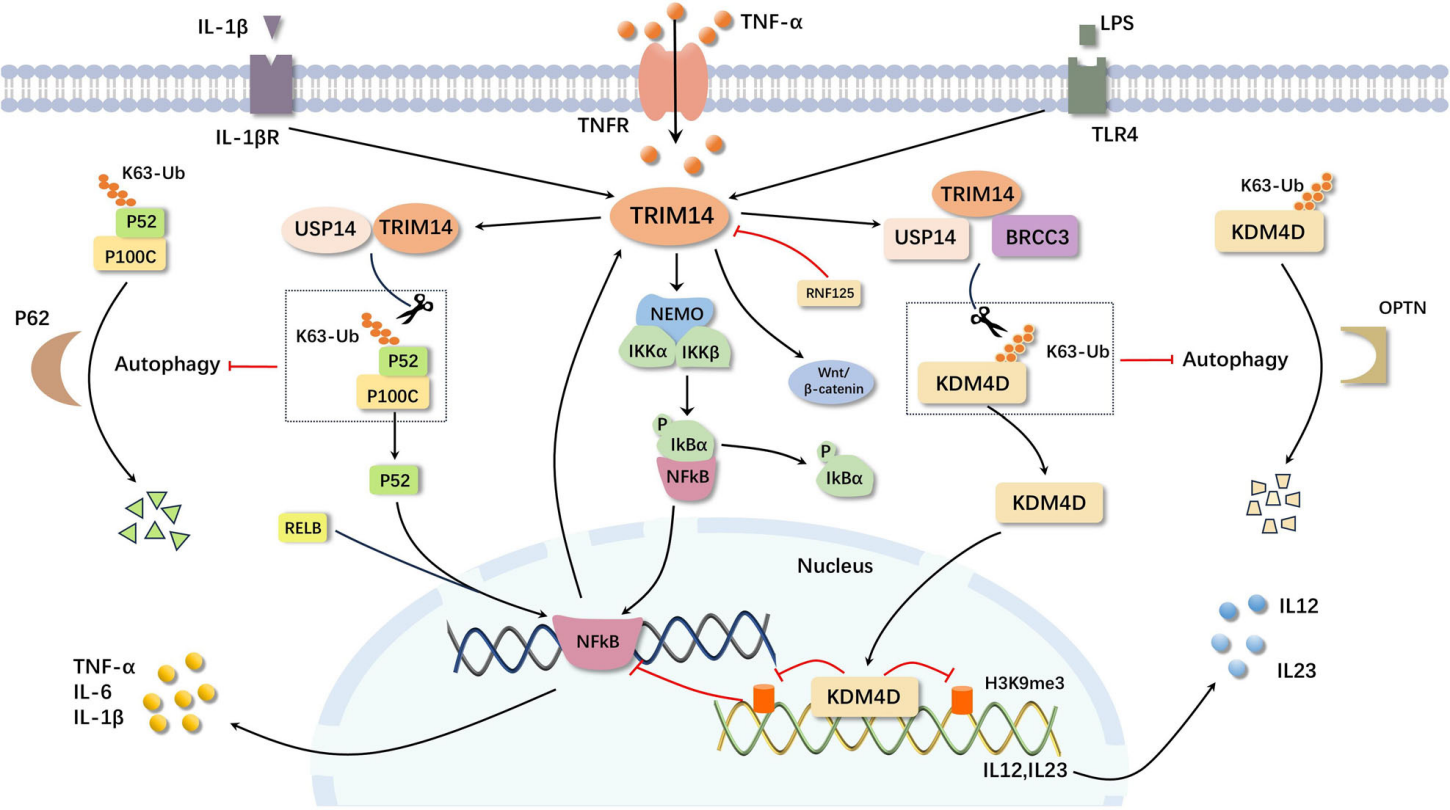
The expression of TRIM14 is upregulated in response to inflammatory stimuli, subsequently enhancing the expression of pro-inflammatory factors. TRIM14 facilitates the progression of inflammation via NF-κB and Wnt/β-catenin signaling pathways, modulates autophagy processes through interactions with USP14, regulates the activation of atypical NF-κB signaling pathways, and functions as an epigenetic regulator that influences gene transcription and autoimmune inflammation.

TRIM14 also plays an increasing role in the inflammatory response. Inflammatory stimuli significantly induce TRIM14 expression, and the presence of TRIM14 also contributes to enhanced expression of inflammatory cytokines [79] (Fig. 4).

#### Chronic periodontitis

In chronic periodontitis, it has been reported that TRIM14 deficiency leads to decreased osteoclast formation and p100 and p52 expression, which in turn inhibits atypical NF-κB signaling pathway activation, thereby preventing bone loss and destruction during chronic periodontitis [87]. Therefore, targeting TRIM14 may be an effective treatment for preventing chronic periodontitis.

#### Osteoarthritis (OA)

As an important regulator of chondrocyte homeostasis in adult articular cartilage, NFATC2 can bind to RNF125 promoter, promote the expression of RNF125, degrade TRIM14, and inhibit the nuclear translocation of β-catenin, thus inhibiting the activation of the Wnt/β-catenin signaling pathway, which enhances cartilage disintegration, chondrocyte hypertrophy, and matrix protease expression. However, overexpressed TRIM14 can effectively reverse the effect of RNF125 on OA progression [88–91]. Moreover, TRIM14 promotes the expression of proteins related to the NF-κB signaling pathway in OA, activates the innate immune response, increases the expression of IFN-β, induces an imbalance in intra-articular homeostasis, and promotes the progression of OA [92]. In summary, TRIM14 promotes OA by participating in the NF-κB pathway and the Wnt/β-catenin pathway, providing new insights into the pathogenesis of TRIM14-regulated OA.

#### Autophagy

Autophagy plays a crucial role in the interplay between immune and inflammatory responses [93]. Mechanistic investigation has demonstrated that TRIM14 interacts with p100/p52 via its PRYSPRY structural domain, inhibiting p62-mediated degradation of p100/p52 autophagy by recruiting USP14 to cleave the K63-conjugated ubiquitin chain at the K332/338/341 loci of p100/p52, thereby facilitating activation of the atypical NF-κB pathway [94]. Furthermore, TRIM14 functions as an epigenetic regulator by recruiting USP14 and BRCC3 to eliminate the K63-conjugated ubiquitin chain from histone demethylase KDM4D, thus preventing autophagic degradation resulting from KDM4D’s interaction with the cargo receptor OPTN. Additionally, the deletion of TRIM14 diminished H3K9me3 removal from IL-12 and IL-23 promoters, consequently protecting mice from autoimmune inflammation [95, 96]. These findings indicate that the TRIM14-USP14 axis is crucial in regulating the atypical NF-κB signaling pathway. They highlight the interaction between autophagy and innate immunity and offer a new strategy for designing fusion proteins that can regulate selective autophagy.

### RNAs associated with the regulation of TRIM14 expression

microRNAs (miRNAs), long non-coding RNAs (lncRNAs), and circular RNAs (circRNAs) are the three major classes of non-coding RNAs. miRNAs typically target and regulate the expression of specific genes, while lncRNAs and circRNAs can negatively regulate miRNAs by sponging them [97–99]. In the context of TRIM14 expression regulation, miRNAs often inhibit its effects by down-regulating TRIM14. In contrast, the corresponding lncRNAs and circRNAs can positively regulate the expression and function of TRIM14 by interacting with miRNAs (Table 1). For example, in CRC, miR-191-5p can down-regulate the expression of TRIM14. At the same time, lncRNA ElNF1-AS1, which is up-regulated due to hypoxia, can positively regulate the expression of TRIM14 through sponging miR-191-5p, thus exerting the cancer-promoting effect of TRIM14 [100]. In addition, circRNA hsa_circ_0060927 may upregulate TRIM14 by sponging miR-195-5p, which in turn promotes the conversion of oral leukoplakia to OSCC [59]. These RNAs are expected to be strategies for artificially regulating TRIM14 expression.

### The antipathogenic role of TRIM14

Studies have shown that TRIM family proteins play an important role in the body’s anti-pathogen immune response. For example, TRIM5α is effective in inhibiting retrovirus infection, including HIV-1 [101]. At the same time, TRIM32 can be involved in the intracellular response of macrophages to mycobacterium tuberculosis infection, thus promoting autophagy-mediated mycobacterium tuberculosis degradation [102]. For TRIM14, it can help the body fight against viruses and bacteria through IFN and related signaling pathways, and it can also directly interact with viral nucleic acids or related proteins, thus becoming a direct antiviral limiting factor against a variety of viruses (Table 2).

**Table 2:** Mechanism of anti-pathogen activity of TRIM14.

Pathogen	Mechanism	References
EBOV	TRIM14 interacts with EBOV’s NP to promote the expression of NF-κB and IFN-β, and inhibit the infection and replication of EBOV.	[127]
IAV	The PRYSPRY domain of TRIM14 directly interacts with IAV’s NP, inducing K48 ligand ubiquitination and proteasome degradation of NP, inhibiting IAV infection and proliferation.	[129, 130]
HBV	TRIM14 interacts with the HBV HBx protein via its PRY-SPRY domain to inhibit the formation of the Smc-HBx-DDB1 complex, thereby suppressing HBV replication.	[23, 141]
HCV	The PRY-SPRY domain of TRIM14 interacts with NS5A of HCV, resulting in ubiquitination dependent degradation of NS5A in a K48-linked manner, inhibiting HCV infection and replication.	[24]
SINV	Enhanced expression of TRIM14 activates the transcription of many immune genes, such as IFN, and inhibits the reproduction of SINV.	[142]
M. tuberculosis	TRIM14 promotes STAT3 phosphorylation through TBK1, negatively regulates the expression of IFN- I in macrophages to avoid the damage to the host caused by overexpression of IFN- I.	[128]
Lm	TRIM14 directly interacts with Lm through its intrinsic antibacterial activity to impede Lm infection.	[131]

### TRIM14 exerts its effects by interacting with interferons (IFNs) and their associated signaling pathways

The immune system can respond to microbial infections with three types of interferons: type I, type II, and type III IFNs. Viral nucleic acids in infected cells lead to the synthesis and secretion of IFN-I, the production of which is one of the initial defense mechanisms of the immune response [103]. At the same time, IFN is also involved in anti-bacterial immune responses, such as IFN-γ secreted by bone marrow-derived macrophages to control intracellular bacterial infections [104, 105].

TRIM14 is induced by type I interferon and play a key role in controlling viral infections [106, 107]. IRFs are a class of transcriptional regulators of IFN and IFN-induced genes that can regulate related genes by directly binding to the interferon-stimulated response elements (ISREs) of ISG promoters [108]. ISRE in the TRIM14 promoter is necessary for IFN-I to regulate TRIM14. Moreover, IRF-1 and IRF-2 can bind to ISRE and mediate TRIM14 transcriptional activation at various stages [109]. RNF125, on the other hand, acts as an E3 ubiquitin ligase that can lead to ubiquitination and degradation of the TRIM14 protein, acting as a negative regulator of the innate antiviral immune response to prevent immune overactivation [110].

Moreover, TRIM14, as a mitochondrial adapter protein, offers a docking platform for the assembly of mitochondrial signaling complexes for maximizing the activation of RIG-I-mediated signaling pathways. The mitochondrial complex WHIP-TRIM14-PPP6C interacts constitutively with MAVS through TRIM14. MAVS interacts with RIG-I in an inducible manner. In uninfected cells, RIG-I is maintained in an inactive state through the interaction of the C-terminal inhibitory domain with the N-terminal caspase activation and recruitment domains (CARDs) and the constitutive phosphorylation of its CARDs by protein kinase C. When viral RNA binds to the ligase domain of RIG-I, CARDs are released, the conformation of RIG-I changes, and the ubiquitin-binding domain (UBD) in WHIP translocates RIG-I to the MAVS mitochondrial platform by binding to the K63 multiprotein chain at RIG-I K164. Upon binding to RIG-I, MAVS recruits various downstream molecules and further activates two kinase complexes: the non-classical IκB kinase (TBK1/IKKi) complex and the classical IKK complex. The TBK1/IKKi kinase phosphorylates IFN regulator 3/7, which translocates to the nucleus and drives IFN transcription. The classical IKK complex releases NF-κB into the nucleus and stimulates the expression of pro-inflammatory genes, which cooperate with IFN to induce an antiviral immune response. The ATPase domain of WHIP contributes to the stabilization of the RIG-I-dsRNA complex and promotes the ubiquitination of RIG-I K63. PPP6C in the WHIP-TRIM14-PPP6C complex dephosphorylates RIG-I, maintains RIG-I activation, and leads to the amplification of downstream antiviral signaling [111–118].

In addition, another study has shown that after the signal bodies containing MAVS and TRIM14 are assembled in the mitochondrial outer membrane, TRIM14 recruits NEMO via a K63-linked polyubiquitin chain, which attaches NEMO to the MAVS signalosome and further activates two NEMO-containing protein kinase complexes, the NEMO-TBK1-IKKi complex and the NEMO-IKKα-IKKβ complex, followed by the activation of IFN regulatory factor 3/7 and NF-κB, respectively, promoting the antiviral response [119].

cGAS not only participates in the occurrence and development of tumors, but also acts as a DNA sensor in innate immune response. When pathogens with DNA infect host cells, cGAS can recognize foreign DNA and be activated, and then catalyze the synthesis of cytoplasmic ATP and GTP into the second messenger molecular ring GMP-AMP (cGAMP). Subsequently, cGAMP activates STING, a transmembrane protein located in the endoplasmic reticulum, which is transported from the endoplasmic reticulum to the Golgi via COPII-mediated vesicles [120–123]. Activated STING recruits and activates TBK1 and IKKβ to promote IRF3 and NF-κB entry into the nucleus, respectively, and ultimately produces IFN-I and proinflammatory cytokines and subsequently initiates an innate immune response [124, 125]. TRIM14 is upregulated by IFN-I, as an ISG-expressed protein, in this process and inhibits the p62-cGAS interaction by recruiting USP14 to cleave the K48-linked ubiquitin chain of cGAS at the K414 site, thereby inhibiting p62-dependent selective autophagic degradation of cGAS, which in turn promotes IFN-I signaling [126].

**Fig. 5:**
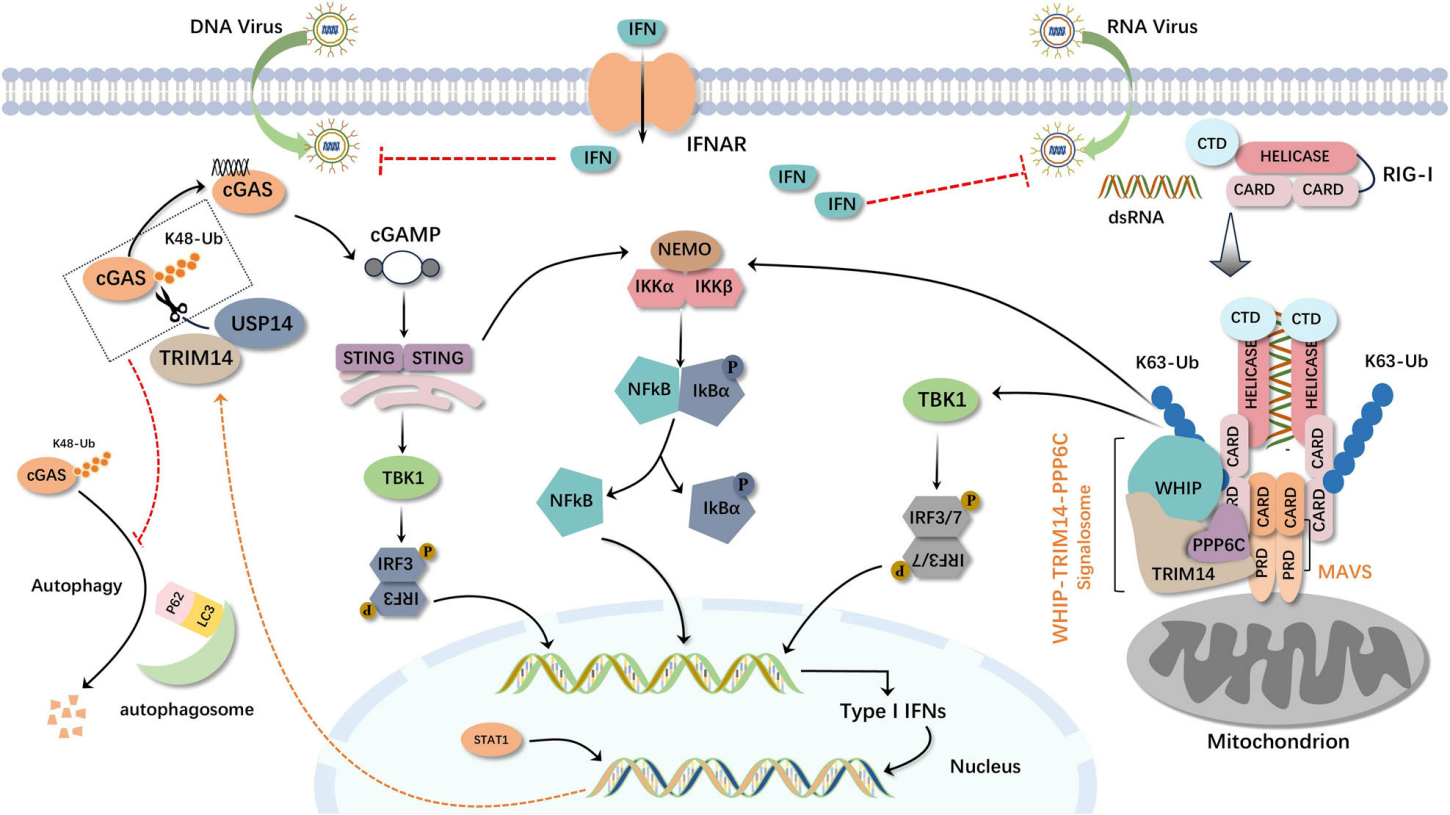
TRIM14 can be stimulated not only by IFNs but also participates in activating the NF-κB signaling pathway through RIG-I/MAVS and cGAS/STING axes, thereby promoting IFNs expression and establishing a reciprocal interaction with IFNs, thus exerting significant antiviral activity.

The interaction of TRIM14 with IFNs and its related signaling pathways is reflected in the immune system’s defense against a spectrum of pathogens. For example, upon infection with the Ebola virus (EBOV), intracellular TRIM14 can interact with EBOV nuclear protein (NP) to promote TRIM14-mediated IFN-β and NF-κB promoter activity to play an anti-EBOV role. Furthermore, TRIM14 does not cause degradation of the EBOV nuclear protein (NP) during this process [127]. Moreover, it is noteworthy that TRIM14 is implicated as a critical negative regulator in the type I IFN response during Mycobacterium tuberculosis infection, thereby avoiding over-induction of type I IFN and reducing the harm to the host [128] (Fig. 5).

The above studies indicate that the mutual regulation of TRIM14, IFNs and its related signaling pathways plays an important role in anti-pathogen immunity. And TRIM14 has the potential to be used as an adjunct to IFNs therapy.

### TRIM14 interacts directly with pathogen components

TRIM14 primarily binds to pathogen components through its PRY-SPRY domain. Upon infection with influenza A virus (IAV), TRIM14 facilitates the ubiquitination and proteasomal degradation of nucleoprotein (NP) via direct interaction between its PRY-SPRY domain and NP, effectively obstructing the translocation of NP from the cytoplasm to the nucleus and thereby limiting IAV replication within host cells. Besides, this antiviral effect may occur in the absence of cytokine storms [129, 130]. A similar mechanism is observed during hepatitis C virus (HCV) infection, where TRIM14’s PRY-SPRY domain interacts with HCV non-structural protein NS5A, promoting K48-linked ubiquitination-dependent degradation of NS5A, which in turn inhibits HCV infection and replication [24]. Besides, during HBV infection, STAT1 can bind to the TRIM14 promoter; consequently, the induced TRIM14 interacts with HBx through its PRY-SPRY domain to inhibit the formation of the Smc-HBx-DDB1 complex, thus diminishing HBx’s role in HBV [23].

Furthermore, one study indicated that during Listeria monocytogenes (Lm) infection, TRIM14 exerts a direct antibacterial effect within host cells; however, this antibacterial activity does not stem from any specific domain within TRIM14 but rather arises from its intrinsic antibacterial properties [131].

These results indicate that TRIM14 can directly act on pathogens and exert anti-pathogen effects. Promoting the expression of TRIM14 may be a strategy to enhance the anti-infection ability of the body.

## Conclusions and perspectives

As research on ubiquitination-related molecules deepens, the role of TRIM14 has gradually emerged. Previous studies have shown that it can promote or inhibit different types of cancer and participate in the development of other diseases and the body’s immune response to pathogens through various pathways. Some of these functions have been proven to be related to ubiquitination or deubiquitination. At the same time, these studies have also identified many regulatory factors associated with TRIM14 expression.

However, a complete understanding of TRIM14’s function remains a missing piece of the puzzle. On one hand, similar to its dual role in the development of cancer, does TRIM14 play diverse or even opposite roles in different diseases? Which domains of the TRIM14 molecule are responsible for which specific functions? On the other hand, although previous studies have revealed the mechanisms of TRIM14 in many pathophysiological processes, providing many new ideas for disease treatment, it should be noted that the clinical translation of TRIM14 research findings is rarely carried out. We believe that further research and exploration of these issues will help to better understand and utilize the functions of TRIM14, improving and perfecting the clinical treatment of related diseases.

## Data Availability

All datasets and materials used in this study are included within the manuscript and available from the corresponding author upon reasonable request.
